# Direct His-bundle Pacing in a Patient with a Persistent Left Superior Vena Cava

**DOI:** 10.19102/icrm.2019.100503

**Published:** 2019-05-15

**Authors:** Amrish Deshmukh, Tasha Wadsworth, Pramod Deshmukh

**Affiliations:** ^1^Department of Internal Medicine, Division of Cardiovascular Medicine, University of Michigan Samuel and Jean Frankel Cardiovascular Center, Ann Arbor, MI, USA; ^2^Department of Internal Medicine, Division of Cardiology, Robert Packer Hospital, Sayre, PA, USA

**Keywords:** Atrioventricular node, catheter ablation, His bundle pacing, persistent left superior vena cava

## Abstract

We report a case of direct His-bundle lead placement at the time of implantable cardioverter-defibrillator insertion and atrioventricular node ablation. The patient was found to have an isolated persistent left superior vena cava, and selective His-bundle pacing was successfully achieved through the use of a steerable sheath and dedicated mapping catheter.

## Case presentation

A 79-year-old male with a history of nonischemic cardiomyopathy, previous bioprosthetic aortic valve replacement, and long-standing persistent atrial fibrillation (AF) presented with syncope. He showed New York Heart Association (NYHA) class IV heart failure symptoms and had an ejection fraction of 10% to 15% despite optimal guidelines-directed neurohormonal blockade. Telemetry monitoring revealed episodes of AF with rapid ventricular rates, which degenerated into nonsustained ventricular tachycardia despite maximally tolerated β-blocker therapy. As the patient had a normal QRS complex, a direct His-bundle pacing with atrioventricular (AV) nodal ablation approach was chosen in order to achieve rate control and maintain physiologic activation of the ventricles. Dual-chamber implantable cardioverter-defibrillator (ICD) placement was planned following consideration of the patient’s long-standing cardiomyopathy.

**Figure 1:**
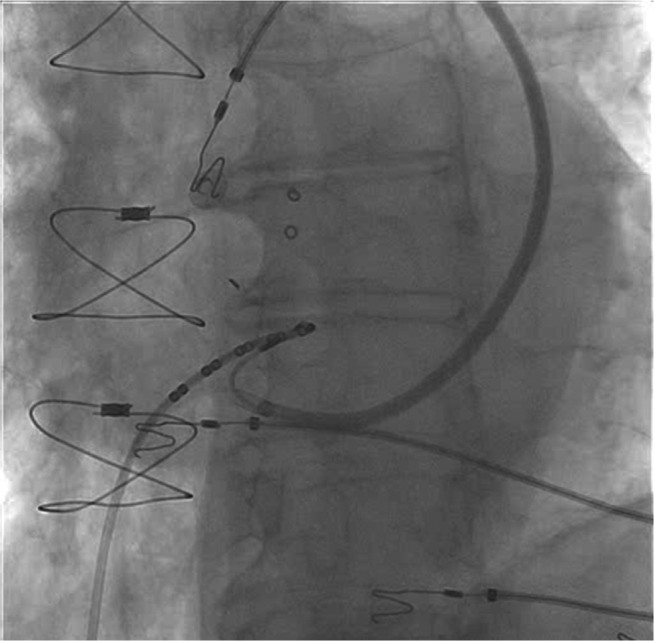
Fluoroscopy demonstrating the use of a right femoral hexapolar catheter as an anatomic guide for the pacemaker lead tip, which is positioned via a steerable catheter passing through the PLSVC, coronary sinus, and right atrium.

**Figure 2:**
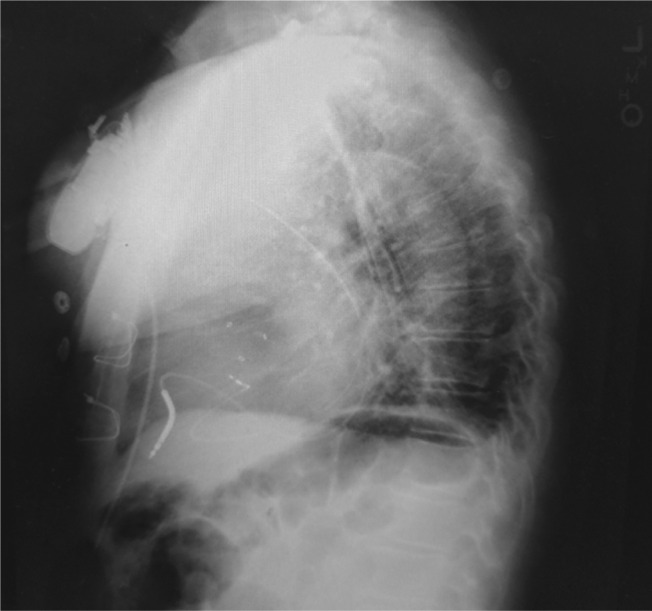
Chest radiograph demonstrating the final His-bundle and ICD lead positions.

**Figure 3:**
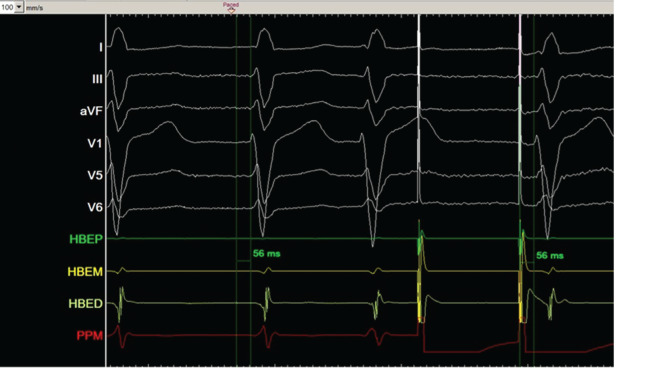
Surface and intracardiac electrocardiograms demonstrating identical H to V and pace to V intervals and the identical morphology of the native and paced QRS complexes.

A right femoral hexapolar catheter was used to map the His bundle. Left subclavian access was obtained and the lead was advanced. Venography of the left subclavian vein suggested the presence of a persistent left superior vena cava (PLSVC) with dilated coronary sinus **(Video 1)**. Therefore, right subclavian access was obtained and venography demonstrated atresia of the right superior vena cava with isolated PLSVC **(Video 2)**. A steerable sheath was introduced into the right subclavian vein and was passed successfully from the PLSVC to the coronary sinus and the right atrium. Using the hexapolar catheter as an anatomic guide, the steerable sheath was subsequently manipulated in order to position the lead along the perceived path of the His bundle **(Figure 1)**. A Medtronic 3830 steroid eluting bipolar active fixation lead (Medtronic, Minneapolis, MN, USA) was employed to record and pace the His bundle **(Figure 2)**. The criteria for direct His-bundle pacing were verified and were as follows: concordance of the paced and native 12-lead electrocardiograms, identical pace–ventricular and His–ventricular intervals, and abrupt loss of capture without QRS widening and with sequentially lowered pacing output **(Figure 3)**. An ICD lead was placed in the right ventricular apex. The His threshold at implant was 1.75 V with an impedance of 430 Ω. The AV node was then mapped and ablated. The total procedure time was 70 minutes, including 30 minutes of fluoroscopy.

At three months after surgery, the His-bundle threshold was 1.0 V with an impedance of 410 Ω. The patient presented NYHA class II heart failure symptoms and an improvement in ejection fraction to 40%. No episodes of nonsustained ventricular tachycardia were recorded during follow-up.

## Discussion

The presence of an isolated PLSVC presents an additional but surmountable challenge in the placement of a permanent pacemaker lead when anatomic selectivity, such as His-bundle pacing, is desired. PLSVC is estimated to be present in 0.5% of both the general population and in patients undergoing permanent pacemaker implantation.^1,2^ Considering patients with PLSVC, approximately 10% have an absent or atretic right superior vena cava.^2,3^ PLSVC results from an abnormal persistence of the left anterior cardinal vein, which typically regresses to form the ligament of Marshall. While present in the absence of other apparent anatomic anomalies, the prevalence of PLSVC is more common in patients with other congenital heart diseases, especially situs abnormalities.^4^

Despite nearly two decades of study since the initial description of the feasibility and criteria of permanent direct His-bundle pacing, the widespread utilization of this approach has remained limited as a result of perceived difficulties associated with lead placement and achieving appropriate thresholds.^5–7^ However, with the more recent development of new pacing leads and delivery systems, long-term success rates of more than 80% have since been reported.^8–11^ In addition, multiple authors have lauded the benefits of His-bundle pacing including the maintenance of physiologic ventricular activation, cardiac resynchronization, improvement of hemodynamic function, and avoidance of traversing the tricuspid valve.^5,9,11,12^

The presence of a PLSVC may pose a significant challenge even in instances of routine device implant.^1,13,14^ The present case demonstrates that, with the use of newer active fixation leads and a steerable sheath, it is possible to achieve direct His-bundle pacing in this setting.^8^ Although many authors seem to perform the procedure without the use of a dedicated mapping catheter, this case highlights the value of considering an anatomic landmark that allowed for the delivery of the pacing lead after making a 360° turn in multiple planes.^9,10^ It is possible that, with the use of three-dimensional electroanatomic mapping, fluoroscopy and procedure times could be further reduced. While this approach has been previously demonstrated in a report of His-bundle pacing at the time of AV nodal ablation, it remains to be studied systematically.^15^

The present case additionally highlights the use of a “pace and ablate” strategy in patients with AF and dilated cardiomyopathy. While typically performed in patients with AF and symptoms or rapid rates that are refractory to pharmacologic therapy, as was true in this patient, or in order to optimize cardiac resynchronization therapy, AV node ablation with His-bundle pacing may also be beneficial for use in patients with controlled ventricular rates.^16,17^ Such an approach employing His-bundle pacing has been shown to result in improvements in functional status and echocardiographic parameters in patients with either reduced or preserved ejection fraction.^18^

Traditional biventricular pacing with left ventricular stimulation via a lead located in the coronary sinus has also been incorporated into patients with PLSVC. However, this approach may be impaired by the limited availability of sufficiently lateral coronary sinus tributaries.^19^ In addition, in patients with narrow QRS complexes, biventricular pacing may induce dyssynchrony and worsen outcomes.^20^ Finally, in acute studies of patients with narrow QRS complexes with AV nodal disease, His-bundle pacing has been demonstrated to have a superior hemodynamic response in comparison with biventricular pacing.^21^ His-bundle pacing was therefore chosen for application in this case to preserve the physiologic activation of the ventricles while achieving rate control.

In summary, with the availability of newer leads and delivery systems, permanent direct His-bundle pacing is feasible, even in the presence of challenging venous anatomy.

## Figures and Tables

**Figure:** Video 1: Cine fluoroscopy of venography obtained from a catheter introduced at the right subclavian vein, which demonstrates an isolated persistent left superior vena that drains into a dilated coronary sinus.

**Figure:** Video 2: Right subclavian access demonstrated right superior vena cava atresia with isolated PLSVC.
